# K8sSim: A Simulation Tool for Kubernetes Schedulers and Its Applications in Scheduling Algorithm Optimization

**DOI:** 10.3390/mi14030651

**Published:** 2023-03-13

**Authors:** Shilin Wen, Rui Han, Ke Qiu, Xiaoxin Ma, Zeqing Li, Hongjie Deng, Chi Harold Liu

**Affiliations:** School of Computer Science and Technology, Beijing Institute of Technology, Beijing 100081, China

**Keywords:** Kubernetes, scheduling algorithms, Kubernetes simulator, real cluster traces

## Abstract

In recent years, Kubernetes (K8s) has become a dominant resource management and scheduling system in the cloud. In practical scenarios, short-running cloud workloads are usually scheduled through different scheduling algorithms provided by Kubernetes. For example, artificial intelligence (AI) workloads are scheduled through different Volcano scheduling algorithms, such as GANG_MRP, GANG_LRP, and GANG_BRA. One key challenge is that the selection of scheduling algorithms has considerable impacts on job performance results. However, it takes a prohibitively long time to select the optimal algorithm because applying one algorithm in one single job may take a few minutes to complete. This poses the urgent requirement of a simulator that can quickly evaluate the performance impacts of different algorithms, while also considering scheduling-related factors, such as cluster resources, job structures and scheduler configurations. In this paper, we design and implement a Kubernetes simulator called K8sSim, which incorporates typical Kubernetes and Volcano scheduling algorithms for both generic and AI workloads, and provides an accurate simulation of their scheduling process in real clusters. We use real cluster traces from Alibaba to evaluate the effectiveness of K8sSim, and the evaluation results show that (i) compared to the real cluster, K8sSim can accurately evaluate the performance of different scheduling algorithms with similar CloseRate (a novel metric we define to intuitively show the simulation accuracy), and (ii) it can also quickly obtain the scheduling results of different scheduling algorithms by accelerating the scheduling time by an average of 38.56×.

## 1. Introduction

With the fast development of 5G, Internet of Things (IoT) and machine learning (ML) technologies, diverse workloads run in today’s cloud data centers. Representative workloads include big data workloads (e.g., [1,2]), cloud computing workloads (e.g., [3,4,5]), and AI workloads (e.g., [6,7,8,9]). Nowadays, Kubernetes [10] has become a prevalent resource management and scheduling framework for the automated deployment, expansion and management of container-based applications. In a real Kubernetes cluster, the workloads’ performance is considerably affected by the resource allocation among them. Note that scheduling algorithms provided by Kubernetes decide how to allocate resources. For a scheduling algorithm, each task is allocated to the node according to its request resources’ amount of each task, instead of its limit resources’ amount. Thus, the request amount determines whether a task can be allocated to the node, and the minimum resource usage of the task on the node. In contrast, the limit amount determines the maximum resource usage of a task on the node.

**Figure 1:**
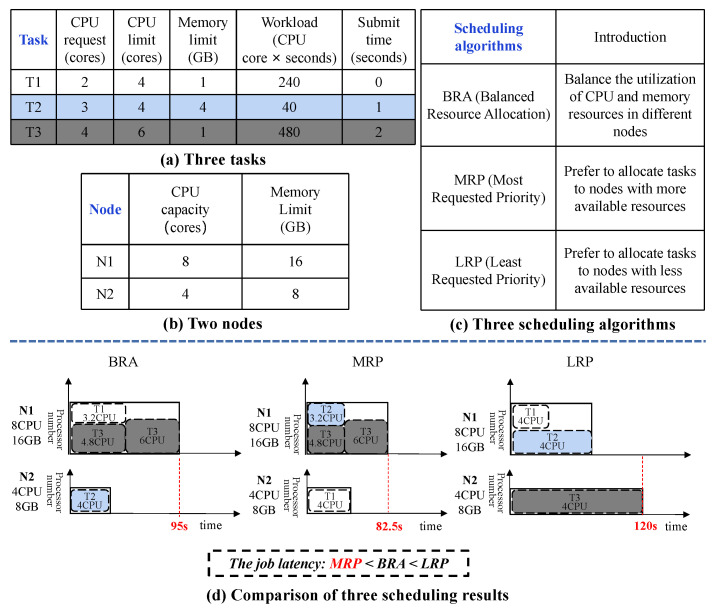
A scheduling example of three tasks in a job using three Kubernetes scheduling algorithms.

**Figure 2:**
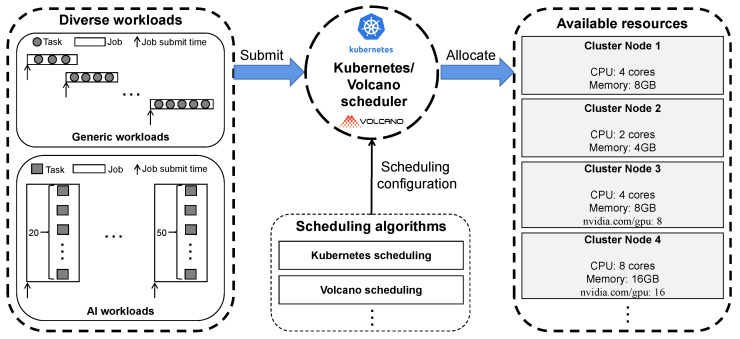
Workload scheduling by using Kubernetes and Volcano schedulers.

**Example** **1.** *Figure 1 illustrates an example of scheduling three tasks (in a job) to two cluster nodes by using three Kubernetes scheduling algorithms. The results demonstrate that the job latency is influenced by three factors: (1) different tasks with requiring different amounts of resources (Figure 1a); (2) available resources of the cluster nodes (Figure 1b); and (3) scheduling algorithms (Figure 1c). When using three different scheduling algorithms, balanced resource allocation (BRA), most requested priority (MRP) and least requested priority (LRP) in Kubernetes [10], the job has considerably different latencies. In addition, Figure 1d shows that compared with the other two algorithms, MRP achieves the lowest latency because its scheduling mechanism can allocate the resources most efficiently for this specific scenario.*

Therefore, in real cluster scheduling, there are two key challenges:

- The selection of scheduling algorithms has considerable impacts on workload performance. Thus, how to accurately evaluate the performance of different scheduling algorithms is a key challenge.
- It takes a prohibitively long time to select the optimal algorithm because applying one algorithm in one single job may take at least a few minutes to complete. Thus, how to quickly obtain the performance of each scheduling algorithm is also a key challenge.

To this end, in this paper, we develop a cluster simulator (based on the popular Kubernetes framework) to effectively and quickly evaluate the performance impacts of different scheduling algorithms. Our contributions are mainly as follows:

▷ **Simulation driven by real-world workload traces.** We study and analyze the real Alibaba cluster traces [11]. Then we obtain the characteristics of real cluster workloads, such as job arrival pattern, the number of tasks in a job, the resource (CPU, GPU and memory) request and resource limit of each task, and the running time of each task. According to this crucial information, we generate two different workloads for effectively evaluating the performance of different scheduling algorithms: TaskQueue workloads and JobQueue workloads.
▷ **Proposed K8sSim framework.** In order to quickly evaluate the performance impacts of different scheduling algorithms, we propose and implement a cluster simulator framework called K8sSim. It have three key components: Http Server, simulation environment and cluster simulator. Among them, the Http Server is used to communicate between the simulation environment and the cluster simulator. The simulation environment is used to provide the information of simulation nodes’ configuration, user-submitted workloads and user-specified scheduling algorithms. The cluster simulator is the core of the whole framework, which is responsible for classifying and scheduling workloads. Most importantly, the cluster simulator implements the Kubernetes simulation scheduler and Volcano simulation scheduler so as to simulate TaskQueue and JobQueue workloads’ scheduling, respectively.
▷ **Evaluation of effectiveness and acceleration of K8sSim.** First, we implement 11 Kubernetes scheduling algorithms and 13 Volcano scheduling algorithms in K8sSim. Then, in order to evaluate its effectiveness and acceleration effect, we conduct a series of experiments in the real Kubernetes cluster and the simulation environment. Note that we define a novel indicator CloseRate to more intuitively show the effectiveness of K8sSim. Finally, the experimental results demonstrate that (i) compared to the real cluster, K8sSim can accurately evaluate the performance of different scheduling algorithms with similar CloseRate, and (ii) by comparing the scheduling times of different algorithms in the two environments, we observe that in all considered scenarios, K8sSim can accelerates this time by an average of 38.56× (acceleration by up to 72.80×).

The remainder of this paper is organized as follows: Section 2 introduces the background and related work. Section 3 describes our proposed cluster simulator framework, and Section 4 evaluates it. Finally, Section 5 summarizes the work.

## 2. Background and Related Work

### 2.1. Background

In modern cloud data centers, a large number of tenants submit a lot of diverse workloads to the cluster. According to the characteristics of these workloads, we roughly divide them into two categories: *Generic workloads* (that is, tasks in a job are submitted to excute sequentially), and *AI workloads* (that is, tasks in a job are executed concurrently by task-group). Figure 2 shows that how the workloads are allocated to cluster nodes by the Kubernetes scheduler or Volcano scheduler. First, a variety of workloads are submitted to the Kubernetes cluster, and then the cluster allocates available resources to these workloads through different scheduling algorithms provided by the Kubernetes or Volcano scheduler. Finally, the workloads are scheduled to run on the cluster nodes.

In this paper, we focus on studying the scheduling of short-running workloads in the Kubernetes cluster.

### 2.2. Existing Benchmark Test Sets

The benchmark test set is a commonly used basis for evaluating cluster performance [2,12], so we study and summarize existing benchmark test sets. Some of them are mainly proposed based on *cloud computing scenarios*, such as (YCSB) [13], Do-cLite [14], CloudSuite [15] and BenchFoundry [16]. The others are mainly proposed based on *edge computing scenarios*, such as RIoTBench [17], AIoTBench [18], EdgeBench [19], EdgeAIBench [20], IoTBench [21], and Defog [22].

Unlike the above benchmark test sets, in this paper, driven by real Alibaba cluster traces [11], we generate two different workloads for effectively evaluating the performance of different scheduling algorithms, thereby effectively comparing the differences between different algorithms. As a result, the generated workloads can provide a basis for evaluating the scheduling algorithms objectively and quantitatively.

### 2.3. Existing Cluster Simulators

In recent years, there has been some research work about job scheduling optimization in various realistic industries. For example, ref. [23] uses a hybrid algorithm of iterated greedy (IG) and simulated annealing (SA) to solve the flexible job shop problem (FJSP) with crane transportation. Considering FJSP with crane transportation and setup times (FJSP-CS), ref. [24] proposes a knowledge-based DQN algorithm to solve FJSP-CS. In addition, ref. [25] proposes a hybrid multi-objective optimization algorithm that combines the distribution estimation algorithm and deep Q-network to solve FJSP. Therefore, the cluster’s performance largely depends on the configurations of scheduling algorithms [26,27,28,29] when workloads dynamically change. To this end, we need to select the optimal one from all the algorithms by comparing the scheduling result of each scheduling algorithm.

However, it needs a rather long time (e.g., 10 min) to obtain the scheduling result of each algorithm in real cluster. Some recent techniques develop simulation platforms to simulate workload working in real systems. For example, ref. [30] proposes DeepEE, a simulation platform based on commercial fluid dynamics software. It can quickly simulate dynamic IT workloads execution and cooling system operation processes. Ref. [31] provides a new generalized and extensible simulation framework called CloudSim, which can support the simulation of network performance in clusters and model the distribution of clouds, service agents, and virtual machines within a data center. Refs. [32,33] designs a simulation platform for predicting system state models, which is constructed using real data logs collected from the Singapore National Supercomputing Center (NSCC). Then these predicted models from the simulation platform are used to rapidly simulate the state evolution of a real data center while the system is running. However, most of them have limitations: (1) their designs target long-running workloads in data centers; and (2) cluster scheduling with a fixed and pre-specified scheduling algorithm. Compared to existing simulators, our proposed simulator is mainly applied to the following scenarios: (1) the design target for short-running workloads in cloud data centers; and (2) multiple different scheduling algorithms as configurable parameters to provide for cluster scheduling.

Hence, in this paper, based on popular Kubernetes framework, we develop a cluster simulator to accurately and quickly simulate workload scheduling in real Kubernetes cluster. In this way, it can be used to quickly evaluate which scheduling algorithm is optimal for different workload scheduling scenarios.

## 3. Our Proposed Cluster Simulator Framework

### 3.1. Overview

**Figure 3:**
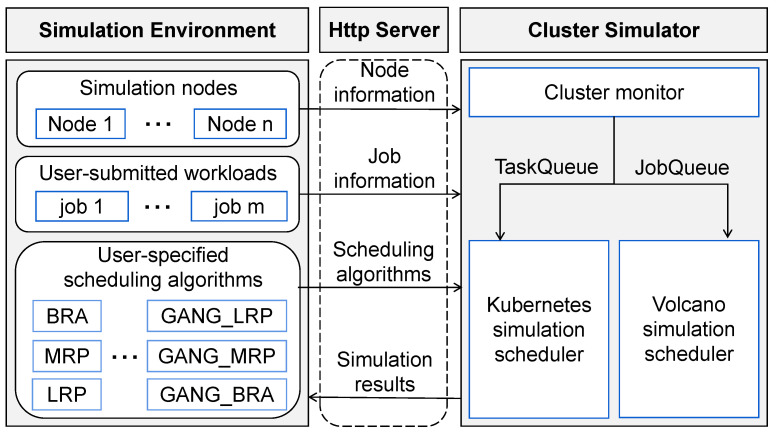
Overview of proposed cluster simulator.

Figure 3 shows the overall design of our proposed cluster simulator software framework and its architectural components. Among these components, the Http Server is responsible for communication between the simulation environment and the cluster simulator.

When the whole framework runs, first the **simulation environment** provides this information to the cluster simulator through Http Server, including the simulation nodes’ configuration, user-submitted workloads and user-specified scheduling algorithms. Next, the cluster monitor of **cluster simulator** receives all information from the simulation environment and initializes the simulation settings (including cluster simulation nodes and waiting for scheduled workloads). At the same time, the monitor parses the workloads’ information into a format (called TaskQueue and JobQueue in this paper) that the simulation scheduler can recognize. Then, these workloads are scheduled by different simulation scheduler according to user-specified scheduling algorithms. For example, the workloads of TaskQueue format (that is, Generic workloads) are scheduled by the Kubernetes simulation scheduler, and other workloads of the JobQueue format (that is, AI workloads) are scheduled by the Volcano simulation scheduler. After the scheduler completes scheduling, the simulator receives the simulation scheduling results. Finally, the simulator returns them to the simulation environment.

Most importantly, our proposed cluster simulator can be deployed on any local machine conveniently. It uses the existing APIs of open-source Kubernetes/Volcano scheduler and re-implements some key classes/functions (including pod classes, node classes, queue classes, clock classes, job/task submission functions and scheduler configuration functions). This ensures the consistency of the interfaces and data between the simulator and the real Kubernetes. In addition, because the life cycle of each task in a job can be simulated in the simulator, the simulator can obtain the status of each node and pod equivalent to the real cluster. At the same time, the simulator can also directly use some scheduling algorithms provided by default in an existing open-source Kubernetes/Volcano scheduler so as to quickly test the impact of each scheduling algorithm on job/task execution. By extending the basic functionalities already exposed to the framework, other users can add or implement new scheduling algorithms based on their own ideas. Other researchers can also perform some tests based on specific scenarios, thereby allowing the development of best practices in all the critical aspects related to cluster scheduling in cloud data center. In general, our proposed simulator is effective, and its scheduling results are relatively accurate, while greatly speeding up the acquisition of scheduling results for different scheduling algorithms.

### 3.2. Http Server

The **Http Server** is an intermediary between the simulation environment and the cluster simulator. When the entire simulation process starts, the cluster simulator first opens a service port, and then the simulation environment starts running the users’ scheduling programs by specifying the corresponding port.

### 3.3. Simulation Environment

In **simulation environment**, there are three important modules: *simulation nodes*, *user-submitted workloads* and *user-specified scheduling algorithms*. They are described in detail as follows.

**Simulation nodes.** The module is responsible for setting up simulation nodes’ information, which can be set by users according to actual requirements. The information consists of some settings for various resources, such as CPU capacity, memory capacity and total GPU number of each node. Note that the current simulation nodes are implemented via user input into a node configuration file, in which the amount of resources for each simulation node is consistent with that in the real cluster. Therefore, the simulation nodes cannot automatically fetch the resource amount of the cluster nodes. In the future, we will continue to improve it by using the existing APIs (e.g., NodeStatus v1 core) provided by Kubernetes to support the automatic fetching feature.

**User-submitted workloads.** The module is responsible for setting up submitted jobs’ information driven by real-world cluster traces [11]. The information includes startTime, taskWorkload, requested and limited resources (such as CPU, memory and GPU) for each task in a job. Similarly, each user-submitted workload is implemented through a configuration file (e.g., Workload.yaml); based on the request and limit resources (such as CPU, Memory, and GPU) for each task in the jobs, we generate a workload configuration file. The simulation environment then transmits it to the simulator via Http Server. Subsequently, when the workload is scheduled in the simulator, the simulator will parse it to obtain information about the workload waiting to be scheduled.

**User-specified scheduling algorithms.** The module is responsible for providing alternative cluster scheduling algorithms for users. These algorithms are implemented in the cluster simulator. They are roughly divided into two categories: *Kubernetes scheduling algorithms* and *Volcano scheduling algorithms*.

For *Kubernetes scheduling algorithms*, there are three typical scheduling algorithms: (1) BRA: this algorithm balances the utilization of CPU and memory resources in different nodes. (2) LRP: this algorithm calculates the amount of resources and the number of tasks allocated to different nodes, and prefers to allocate tasks to nodes with more available resources. (3) MRP: this algorithm prefers to allocate tasks to nodes with less available resources, thus running the same tasks with the least number of nodes.

For *Volcano scheduling algorithms*, there are also three scheduling algorithms: (1) GANG_LRP: this algorithm means that first, only when the cluster resources meet the request of the minimum parallel tasks required by a job, the job can be scheduled (that is, GANG [34]); then it prefers to allocate tasks of the job to nodes with more available resources. (2) GANG_MRP: for this algorithm, first, only when the cluster resources meet the request of the minimum parallel tasks required by a job, the job can be scheduled; then it prefers to allocate tasks of the job to nodes with less available resources. (3) GANG_BRA: this algorithm indicates that first, only when the cluster resources meet the request of the minimum parallel tasks required by a job can the job be scheduled; then it prefers to allocate tasks of the job to nodes with more balanced resources. In addition, DRF [35] and SLA [36] are also two typical allocation algorithms for how jobs are scheduled in the Volcano scheduler.

### 3.4. Cluster Simulator

**Figure 4:**
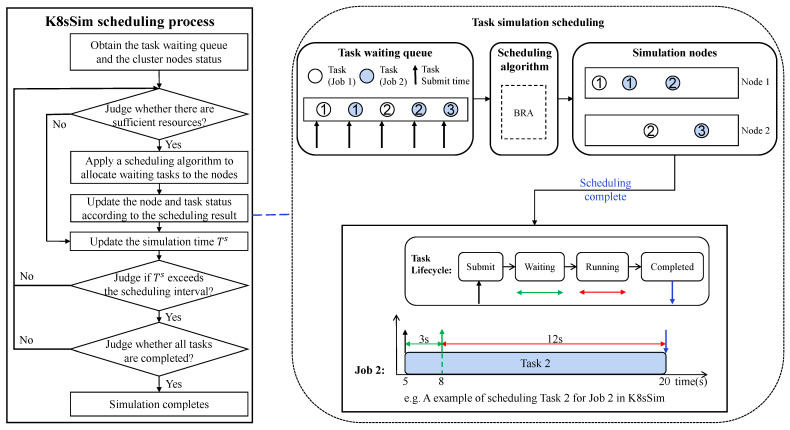
K8sSim-based scheduling process.

**The cluster simulator** is the core of the whole framework, which is responsible for simulating jobs’ scheduling according to the simulation nodes, jobs and scheduling algorithm information provided from the simulation environment. After the scheduling process is completed, the cluster simulator will generate the corresponding scheduling results, and then return them to the simulation environment. In detail, Figure 4 illustrates the simulation scheduling process based on K8sSim. First, the simulator obtains the task waiting queue and the cluster nodes’ status from the simulation environment. Then, for all tasks to be scheduled in the waiting queue, the simulator judges whether there are sufficient node resources. If the available resources exceed the requested resources by the waiting tasks, the simulator applies a scheduling algorithm to allocate the tasks to the simulation nodes, and updates the node and task status; otherwise it updates the simulation time $T^{s}$. Next, the simulator judges if it exceeds the scheduling interval (e.g., 2 min) and whether all waiting tasks are completed. If all tasks have been completed, the simulation completes; otherwise it continues scheduling the remaining tasks.

Note that the key functions implemented by the simulator are as follows: (1) It initializes the workload and node status by parsing the configuration files (including user-submitted workloads and simulation nodes) into the formats (that the simulator can recognize). These configuration files are generated according to the formats required in real Kubernetes cluster scheduling. In addition, the specific configuration information (mainly including request and limit resources of tasks and resources capacity of nodes) in the simulator is consistent with the settings in the real Kubernetes cluster. (2) For a specified scheduling algorithm, the simulator rewrites the scheduler configuration functions and uses the scheduler’s API to directly call some default scheduling algorithms from the existing Kubernetes/Volcano scheduling algorithm library. Thus, the simulator implements the same scheduling mechanism as real Kubernetes. (3) Figure 4 illustrates an example of scheduling five tasks (two tasks of Job 1 and three tasks of Job 2) to two simulation nodes (Node 1 and Node 2) by using BRA. After the simulation scheduling completes, the simulator obtains the simulation results. We can see that for each task in the task waiting queue, the simulator can simulate its entire life cycle, including four phases: *submit*, *waiting*, *running* and *completed*. **For example**, when Task 2 of Job 2 in K8sSim is completed, we can obtain the results of its four scheduling phases: the submission time is 5; the waiting time is 3 s; the time to start allocating resources for execution is 8; and the completion time is 20. Thus, the running time is 12 s, and the total time from submission to completion is 15 s.

**Two metrics.** In this paper, for a job, the ***Job Latency*** represents the completion time of a job. Specifically, let *T* be the set of tasks in a job. Let $TST_{i}$ be the submission time of a task $t_{i}\in T$, and $TCT_{i}$ be the completion time of a task $t_{i}\in T$. So the ***Job Latency*** of a job is

(1) $$JobLatency^{job}=max\left(TCT_{i}\right)-min\left(TST_{i}\right)$$

For the task waiting queue, the ***Makespan*** represents the total time spent from the start of the first job to the end of all jobs. Specifically, let *J* be the set of jobs in the waiting queue. Let $JST_{i}$ be the submission time of a job $j_{i}\in J$ (that is, the minimum task submission time in this job), and $JCT_{i}$ be the completion time of a job $j_{i}\in J$ (that is, the maximum task completion time in this job). So the ***Makespan*** of the task waiting queue is

(2) $$Makespan^{queue}=max\left(JCT_{i}\right)-min\left(JST_{i}\right)$$

In addition, to schedule workloads with different formats, the simulator implements two popular simulation schedulers: ***Kubernetes simulation scheduler*** and ***Volcano simulation scheduler***.

#### 3.4.1. Kubernetes Simulation Scheduler

The function of Kubernetes scheduling simulation process is explained in Algorithm 1. This function first pushes each task in *T* to a TaskQueue, according to the start time of each task (lines 1 to 3). It then applies $Scheduler_{alg}$ (for example, LRP) from the simulation environment (line 4). Then it simulates resource allocations (lines 5 to 10). At each round of scheduling, the function first obtains and pops a task from the TaskQueue through the FIFO method (lines 6 to 7). Next, it sequentially allocates the most suitable node to this task by using scheduling algorithm $Scheduler_{alg}$ and binds this task to the node (lines 8 to 9). The simulation ends when all tasks in the TaskQueue are allocated to the nodes. Finally, the function obtains the final scheduling results (line 11).
**Algorithm 1** Kubernetes scheduling simulation.**Require:**$Scheduler_{alg}$: the scheduling algorithm;   *T*: the set of tasks waiting for being scheduled;   *N*: the set of nodes.  1. **for** each task in T **do**  2.    TaskQueue.Push(task);  3. **end for**  4. TaskQueue.Load($Scheduler_{alg}$);  5. **while** not TaskQueue.Empty() **do**  6.    task ← FIFO(TaskQueue);  7.    TaskQueue.Pop(task);  8.    bindingNode ← Allocate(N, task, $Scheduler_{alg}$);  9.    Bind(bindingNode, task); 10. **end while** 11. **return** GetSchedulingResults().

**Figure 5:**
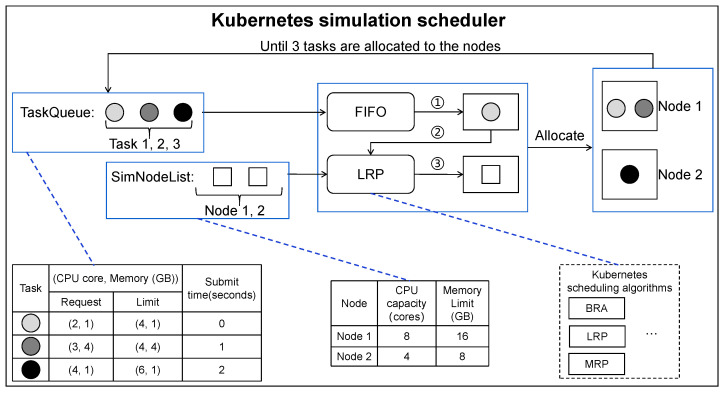
An example of TaskQueue scheduling using LRP in Kubernetes simulation scheduler.

**Example** **2.** *Figure 5 illustrates an example of allocating three tasks (in a TaskQueue) to two simulation nodes, by using LRP which is implemented in the* Kubernetes simulation scheduler. *The results show that when using LRP in this scenario, Task 1 and Task 2 are allocated resources to execute on Node 1, while Task 3 is allocated resources to execute on Node 2. Note that *①* (corresponding to line 7 in Algorithm 1) represents that the simulator tasks out a task from the TaskQueue through FIFO priority, and *②③* (corresponding to line 8 in Algorithm 1) represents that the node is selected from SimNodeList by the allocation mechanism of LRP, according to the status of this task to be executed and SimNodeList.*

#### 3.4.2. Volcano Simulation Scheduler

Similarly, the function of the Volcano scheduling simulation process is explained in Algorithm 2. In detail, this function first pushes each job in *J* to a JobQueue, according to the submission time of each job (lines 1 to 3). It then applies $Scheduler_{alg}$ (for example, GANG_BRA) from the simulation environment (line 4). Then it simulates resource allocations (lines 5 to 20). At each round of scheduling, the function first obtains and pops a job from the JobQueue through the FIFO method (lines 6 to 7). Then it obtains all tasks belonging to the job and sequentially allocates the most suitable node to each task by using scheduling algorithm $Scheduler_{alg}$ (lines 8 to 9). Note that if Gang is used in this scheduling, the function firstly judges how many tasks can be allocated to the nodes. Only when the number of allocated tasks meets the minimum requirements of this job can they actually be scheduled (lines 10 to 14). For example, if a job requires 3 tasks to run at the same time to work properly, while only 2 tasks can be allocated to the nodes in this scheduling, this job will not be scheduled. However, if Gang is not used, these tasks of a job can be directly allocated to the nodes (lines 15 to 17). Next, the function judges that if a job still has some tasks that have not been scheduled to the nodes for execution, the job only with those unscheduled tasks is pushed back into the JobQueue (lines 18 to 20). The simulation ends when all jobs in the JobQueue are allocated to the nodes, and finally the function obtains the final scheduling results (lines 21 to 22).

**Figure 6:**
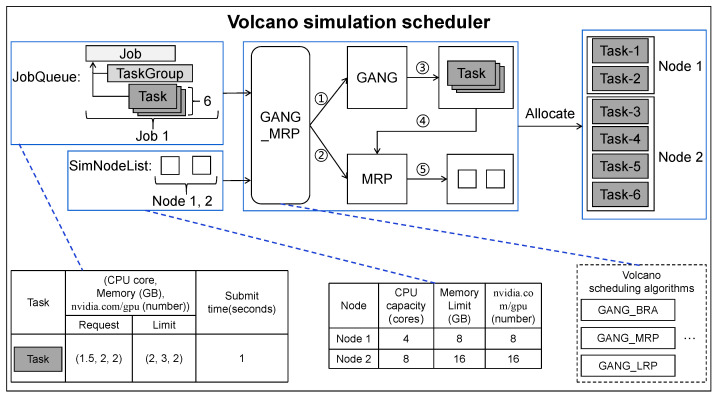
An example of JobQueue scheduling using GANG_MRP in Volcano simulation scheduler.

**Example** **3.** *Figure 6 shows an example of allocating a job with six concurrent tasks (in a JobQueue) to two simulation nodes by using GANG_MRP, which is implemented in* Volcano simulation scheduler. *The results show that when using GANG_MRP in this scenario, two of six tasks are scheduled to run on Node 1, while the remaining tasks are scheduled to run on Node 2. Note that *①②* (line 9 in Algorithm 2) means that after the simulator obtains Job 1, SimNodeList and a scheduling algorithm GANG_MRP, the scheduling process is divided into two steps: (1) First, the simulator will judge whether the cluster resources meet the resource requirements of Job 1 through the allocation mechanism of GANG; *③* (line 12 in Algorithm 2) means if the cluster resources meet the requirement of Job 1, a schedulable TaskGroup of Job 1 will be obtained. (2) Then, *④⑤* (line 13 in Algorithm 2) means that the simulator will assign corresponding simulation nodes to each task in the TaskGroup by the allocation mechanism of MRP, according to the obtained TaskGroup and SimNodeList status.*

**Algorithm 2** Volcano scheduling simulation.**Require:**$Scheduler_{alg}$: the scheduling algorithm;   *J*: the set of jobs waiting for being scheduled;   *N*: the set of nodes.  1. **for** each job in J **do**  2.    JobQueue.Push(job);  3. **end for**  4. JobQueue.Load($Scheduler_{alg}$);  5. **while** not JobQueue.Empty() **do**  6.    job ← FIFO(JobQueue);  7.     JobQueue.Pop(job);  8.    tasks ← Job.GetTasks(job);  9.    NodeTaskPairs←Allocate(N, tasks, $Scheduler_{alg}$); 10.    **if** $Scheduler_{alg}$.hasGang() **then** 11.       **if** NodeTaskPairs.Len()≥Job.RequireNum() **then** 12.          $NodeTaskPairs_{Gang}$←Allocate(N, tasks, $Scheduler_{alg}$); 13.          Schedule($NodeTaskPairs_{Gang}$); 14.       **end if** 15.  **else** 16.       Schedule(NodeTaskPairs); 17.  **end if** 18.  **if** not Job.ScheduleAllTask() **then** 19.       JobQueue.Push(job); 20.  **end if** 21. **end while** 22. **return** GetSchedulingResults().


## 4. Evaluation

In this section, we perform the evaluation experiments. Section 4.1 describes our evaluation settings. Section 4.2 evaluates the effectiveness of our proposed simulator by comparing the scheduling results of two workloads in the simulator and the real cluster. Section 4.3 evaluates the acceleration effect of our proposed simulator (compared to the real cluster).

### 4.1. Evaluation Settings

**Evaluation platform.** For evaluation experiments, we built a Kubernetes cluster, and the specific configuration is as follows:

▷ Intel(R) Xeon(R) CPU E5-2680 v4 @2.40GHz processor, 56 Cores, 256 GB memory (physical machine);
▷ 1 Master Node + 8 Worker Nodes (9 virtual nodes):
  - 16 Cores and 32 GB memory/Master Node, 2 Cores and 4 GB memory/Two Worker Nodes, 4 Cores and 8 GB memory/Four Worker Nodes, and 8 Cores and 16 GB memory/Two Worker Nodes;
  - Linux Ubuntu 18.04 LTS;
  - Python 3.8.5, Go 1.17.6, Docker 20.10.14, Volcano v1.0, and Kubernetes v1.19.0;
▷ AMD Ryzen 7 3700X 8-Core @3.59GHz Processor, 16 GB of DRAM (a machine for conducting simulations).

**Real-trace driven scheduling workloads.** In the evaluation, we generate two workload patterns: *TaskQueue workloads* and *JobQueue workloads*.

▷ For generating *TaskQueue workloads*, the basis is as follows:
  - Driven by Alibaba cluter-trace-v2018 [11] that records the information in the mixed CPU cluster with 4034 nodes running in 8 days;
  - Two typical application scenarios: *Daytime* (6:00 to 24:00) and *Night* (0:00 to 6:00);
  - 19,500 jobs, 6.92 million tasks submitted *in the Daytime*, and 28,300 jobs, 7.00 million tasks submitted *at Night* (in the trace).

▷ For generating *JobQueue workloads*, the basis is as follows:
  - Driven by Alibaba cluter-trace-gpu-v2020 [11] that records the information collected from Alibaba API (artificial intelligence platform) with over 6500 GPUs (about 1800 machines) in a month;
  - Two typical application scenarios: *Daytime* (8:00 to 24:00) and *Night* (0:00 to 8:00);
  - 1,759,052 jobs, 12.54 million tasks submitted *in the daytime*, and 2,462,675 jobs, 17.55 million tasks submitted *at night* (in the trace).

In addition, we also derive some crucial information of these generated workloads from the traces, such as job arrival pattern (here, job is submitted exactly at the job submission interval of the real trace), the number of tasks in a job, the resource (CPU, GPU and memory) request and resource limit of each task, and the workload (that is, the running time of a task).

**Evaluation scenarios and Evaluation metrics.**

In evaluation, we test 20 scenarios, as shown in Table 1. We consider job performance, simulation efficiency and simulation acceleration as the evaluation metrics.

▷ *Job performance*: measured by the average job latency (obtained by calculating the average job latency for multiple jobs in a workload, as shown in Equation (1)).
▷ *Simulation efficiency*: measured by comparing the scheduling results of the simulator and the real cluster (that is, evaluating how close the simulator is to the real cluster).
▷ *Simulation acceleration*: measured by comparing the simulation running time and the real running time of each scheduling algorithm (obtained by calculating the total execution time from the start of the first job to the end of all jobs in a workload, as shown in Equation (2)).

In order to evaluate the simulation efficiency more intuitively, we define an indicator: $CloseRate_{alg}=\frac{AvgJCT(alg)}{min_{alg\in R}AvgJCT(alg)}$, which can be used to show how close each scheduling algorithm is to the optimal. *R* is a set of all algorithms in a certain scenario, AvgJCT(alg) indicates the average job latency of an algorithm, and $min_{alg\in R}AvgJCT(alg)$ indicates the minimum of average job latencies for all algorithms. When $CloseRate_{alg}$ is 1, it indicates that the algorithm is optimal.

### 4.2. Evaluate Effectiveness of Cluster Simulator

**Figure 7:**
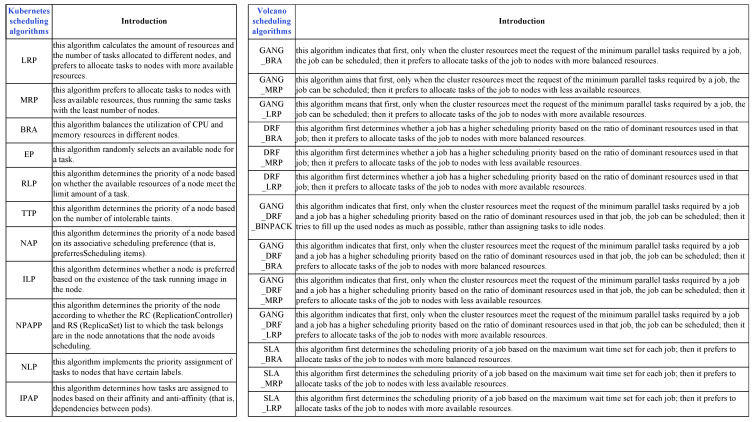
Description of 11 Kubernetes scheduling algorithms and 13 Volcano scheduling algorithms.

**Experimental settings.** In the evaluation, we test four workloads covering generated *TaskQueue* and *JobQueue* workload patterns and two periods (*Daytime* and *Night*). We consider four resource changes: *50% decrease*, *25% decrease*, *25% increase*, and *50% increase* in cluster nodes. In addition, we evaluate 11 representative scheduling algorithms provided by the Kubernetes scheduler [10]: LeastRequestedPriority (LRP), MostRequestedPriority (MRP), BalancedResourceAllocation (BRA), EqualPriority(EP), ResourceLimitsPriority (RLP), TaintTolerationPriority (TTP), NodeAffinityPriority (NAP), ImageLocalityPriority (ILP), NodePreferAvoidPodsPriority (NPAPP), NodeLabelPriority (NLP), and InterPodAffinityPriority (IPAP). Similarly, we also evaluate 13 representative scheduling algorithms provided by the Volcano scheduler [37]: GANG_BRA, GANG_MRP, GANG_LRP, DRF_BRA, DRF_MRP, DRF_LRP, GANG_DRF_BINPACK, GANG_DRF_BRA, GANG_DRF _MRP, GANG_DRF_LRP, SLA_BRA, SLA_MRP and SLA_LRP. As shown in Figure 7, we introduce in detail the functions of all the above scheduling algorithms.

**Figure 8:**
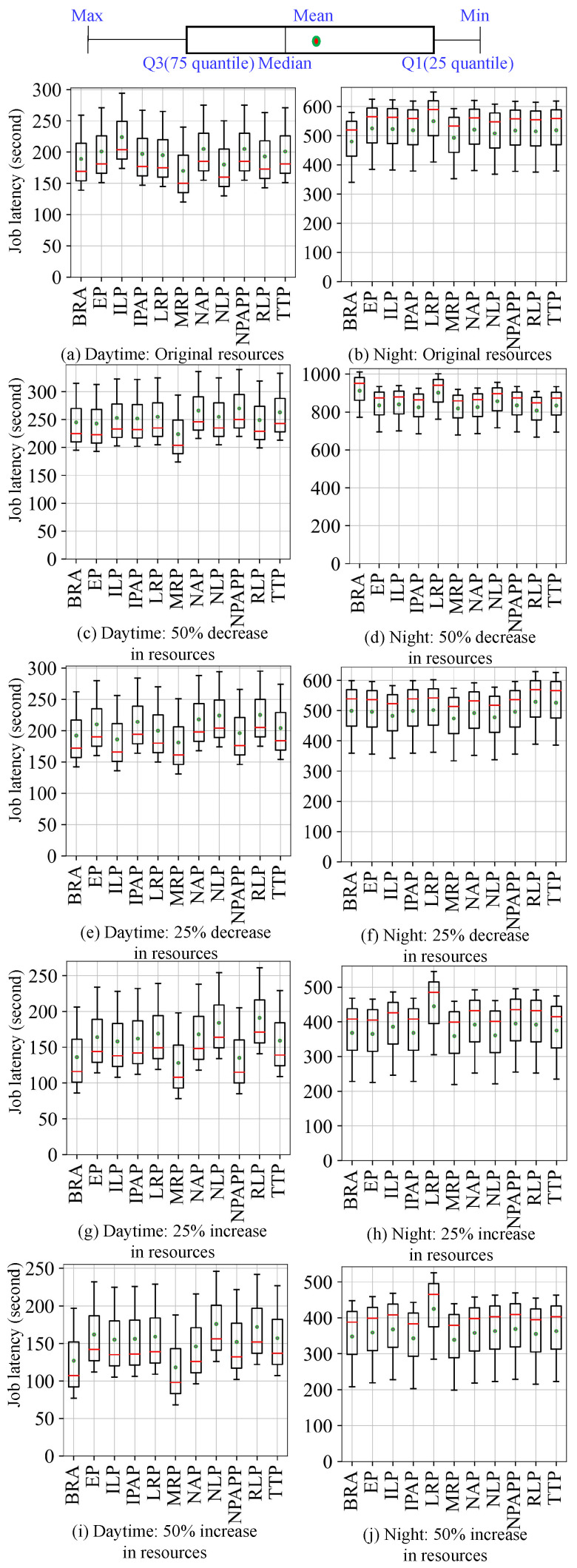
Simulation: TaskQueue workloads.

**Figure 9:**
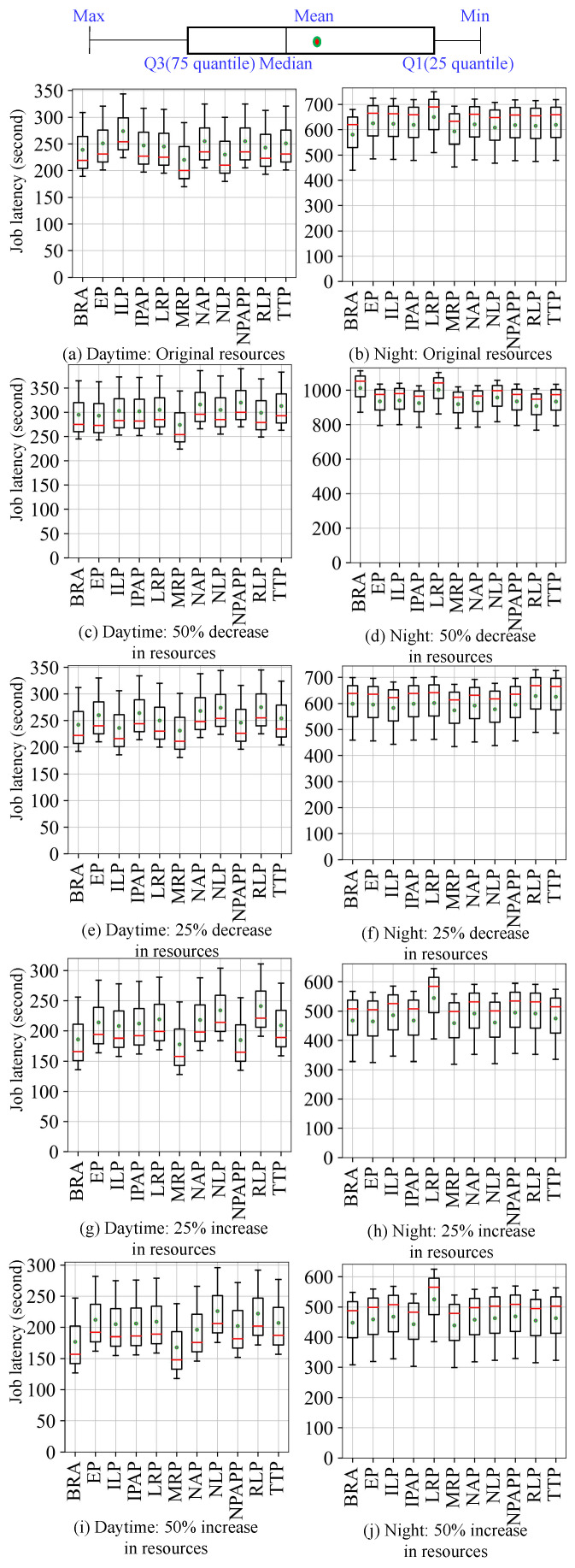
Real cluster: TaskQueue workloads.

**Figure 10:**
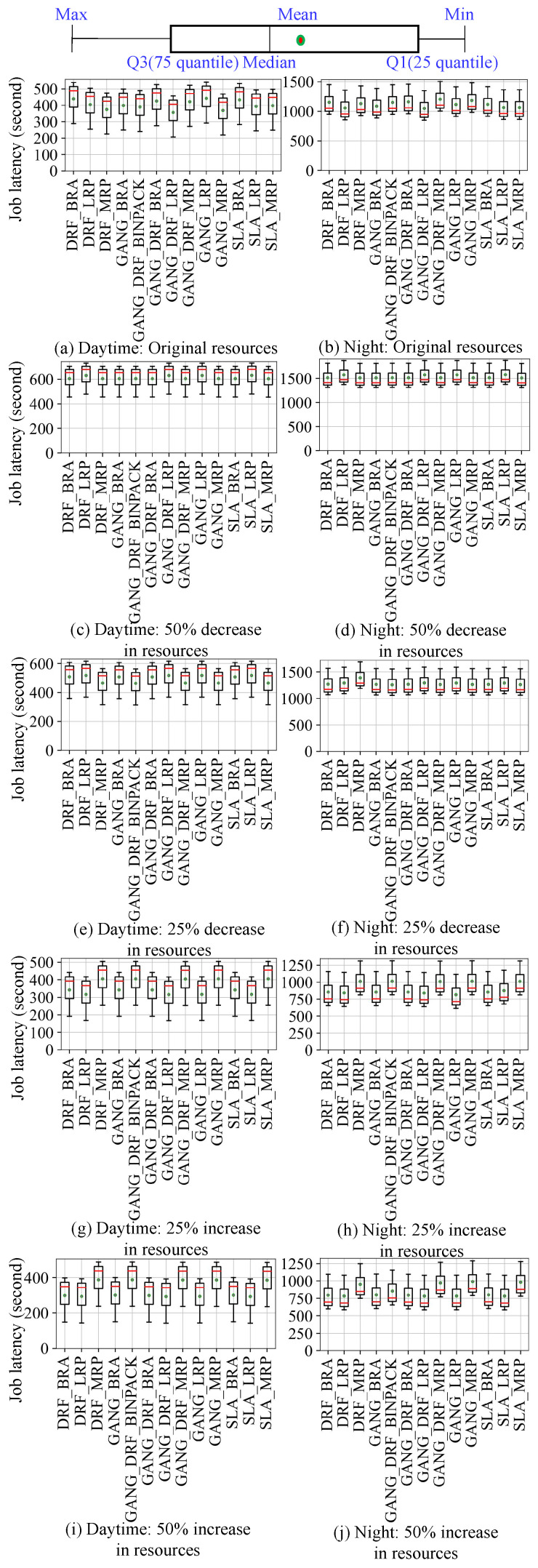
Simulation: JobQueue workloads.

**Figure 11:**
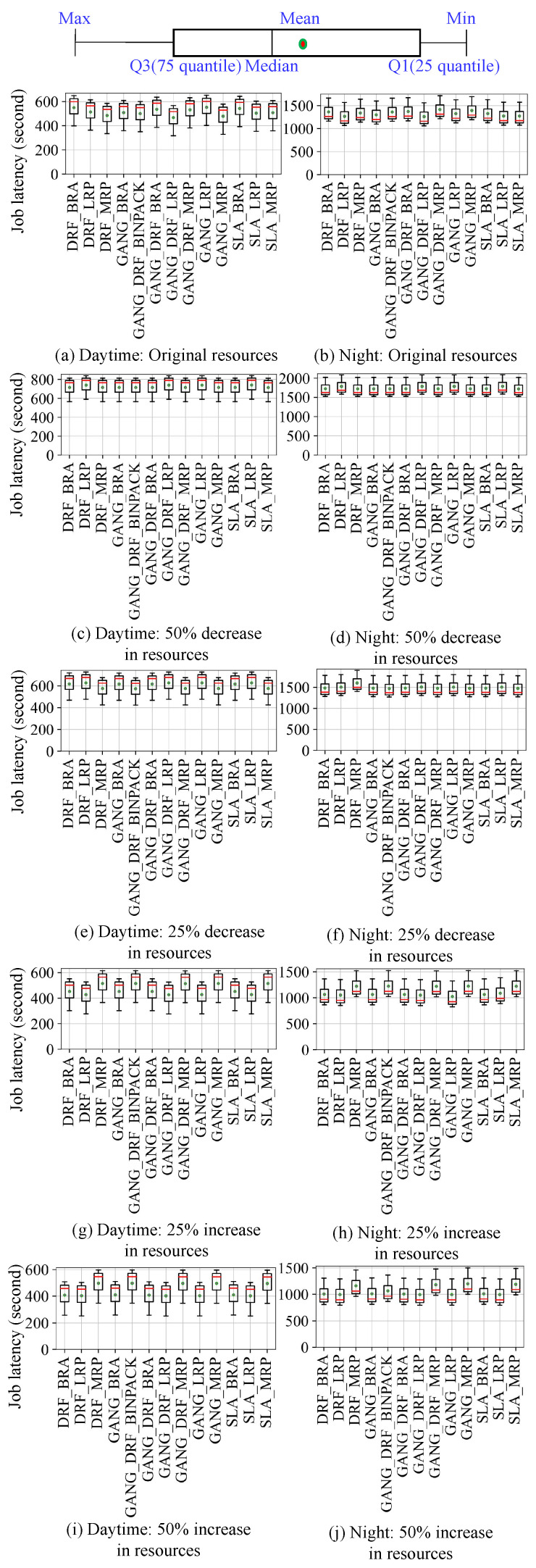
Real cluster: JobQueue workloads.

**Figure 12:**
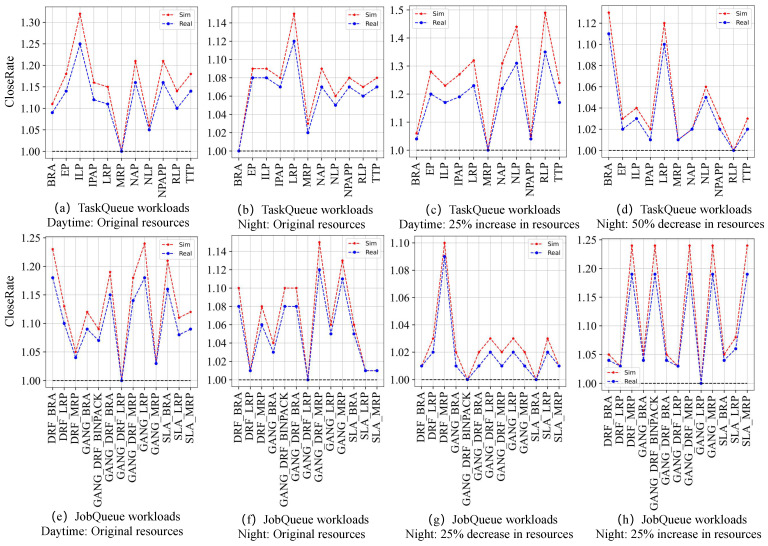
Comparison of CloseRate in simulation and real cluster environments under different workloads and changeable available resources.

Then we submit the four workloads to the simulation and real cluster environments, respectively, so as to obtain the scheduling results in the two environments. As shown in Figure 8, Figure 9, Figure 10 and Figure 11, they use box plots to illustrate each workload’s distribution of job latencies in the two environments. Among them, Figure 8 and Figure 9 show the scheduling results of two TaskQueue workloads in simulator and real cluster, respectively. Similarly, Figure 10 and Figure 11 demonstrate the scheduling results of two JobQueue workloads in the simulator and real cluster, respectively. For example, Figure 8a indicates that under the daytime and original cluster resources, when scheduling the TaskQueue workload in our simulator through 11 Kuberentes scheduling algorithms, we can obtain the simulation scheduling results (that is, the workload’s distribution of job latencies under different scheduling algorithms). Correspondingly, Figure 9a indicates that under the daytime and original cluster resources, when scheduling the TaskQueue workload in the real Kubernetes cluster through 11 Kuberentes scheduling algorithms, we can obtain the real scheduling results. Note that we can see that under the daytime and original cluster resources, the average job latency is minimal when using *MRP* in our simulator and the real cluster, so *MRP* is the optimal scheduling algorithm in both environments. Based on these scheduling results, we compare the difference between the simulation environment and the real cluster environment. In addition, as shown in Figure 12, we select eight groups of scenarios as the demonstration cases to evaluate the simulator’s accuracy, by comparing CloseRate under different workloads (Figure 12a,b,e,f) and changeable resources (Figure 12c,d,g,h), respectively. For example, Figure 12a shows that under the daytime and original cluster resources, when scheduling the same TaskQueue workload through 11 Kuberentes scheduling algorithms, we can obtain the *CloseRate* values of our simulator and real Kubernetes cluster. By comparison, we can see that when the chosen scheduling algorithm is *MRP*, both *CloseRate* values are 1.0, which shows that *MRP* is the optimal scheduling algorithm in both simulation and real environments. On the contrary, compared with other scheduling algorithms, the *CloseRate* value is the largest when using *ILP* in the simulation and real environments, which shows that *ILP* is the worst scheduling algorithm in both environments.

**Experimental results.** As a result, we can observe that on the one hand, compared to the simulator (shown in Figure 8 and Figure 10), the scheduling job latency may be longer in the real cluster environment (shown in Figure 9 and Figure 11). It may be because the use of machines in a real cluster environment is affected by many force-majeure factors, so it is difficult to achieve completely desirable scheduling, thereby leading to longer job latency. On the other hand, we can also see that in most of the scenarios, the performance of each algorithm is basically consistent between the two environments (shown in Figure 12), which proves the effectiveness of the proposed cluster simulator. In addition, we also consider the impact of some uncertainty factors (e.g., persistence of machine usage, and OS activities) on the scheduling results on the real cluster as well as on the simulator. Although the scheduling results are subject to some errors, we can find through extensive testing experiments that in most scenarios, for two scheduling algorithms *A* and *B*, if the job latency of *A* is smaller than *B* on the simulator, then the job latency of *A* is also smaller than *B* when scheduling on the real Kubernetes cluster. This also proves the effectiveness of K8sSim.

In summary, our proposed simulator can achieve a scheduling effect close to the real cluster (that is, the scheduling results have a relatively high accuracy). Therefore, it can be used to accurately evaluate the cluster workloads’ scheduling performance of different scheduling algorithms.

### 4.3. Acceleration of Cluster Simulator

**Experimental settings.** In the evaluation, for different scheduling algorithms, we test the real running time (in the real cluster) and the simulation running time (scheduling by the simulator) under two workloads and changeable resources.

**Experimental results.**Table 2 and Table 3 show the results of real and simulation running time in all considered scenarios. For example, in Scenario 1 (that is, scheduling the TaskQueue workload under the daytime and original cluster resources) of Table 2, when the chosen scheduling algorithm is *BRA*, the real running time in the real Kubernetes cluster is 1868.64 s, while the simulation running time in our simulator is 43.1 s. Table 4 and Table 5 list the reductions of real and simulation running time in these scenarios. For example, in Scenario 1 (that is, scheduling the TaskQueue workload under the daytime and original cluster resources) of Table 4, when the chosen scheduling algorithm is *BRA*, the ratio of the real running time and the simulation running time is 43.36, thus our simulator can accelerate the real running time of *BRA* in the real Kubernetes cluster by 43.36×. More importantly, we can observe that in all scenarios, the running time of workload scheduling in the real cluster is longer (more than 20 min), while the simulator can reduce this time to a few minutes (acceleration by up to 72.80×). When considering all scenarios, we can also see that the simulator can accelerate the running time by an average of 38.56×.

In summary, the simulator can quickly obtain the running results of each scheduling algorithm and compare their performance. Thus, our proposed simulator is more conducive to quickly deciding and selecting the optimal scheduling algorithm for a variety of scenarios (i.e., different jobs, tasks, and nodes), thereby improving the workload performance.

## 5. Conclusions

In this paper, we propose a cluster simulator called K8sSim, a simulation tool for Kubernetes schedulers and its applications in scheduling algorithm optimization. Then we perform a series of experiments to evaluate effectiveness and acceleration effect of K8sSim. The experimental results show that under different workloads and changeable resources, K8sSim can not only ensure the accuracy of scheduling results but also greatly accelerate the scheduling time of different scheduling algorithms in the real cluster. Therefore, our proposed K8sSim can provide a convenient analysis tool and a basis for a series of research works on cluster scheduling optimization.

However, the current K8sSim still has some shortcomings and needs to be further improved in future work. (1) The supported algorithms are still relatively limited and need to be further improved to support more. (2) When the scale of the cluster nodes and workloads is larger (i.e., thousands of nodes and jobs), the scheduling results obtained from the simulator will become less accurate, so further improvement is required to support the larger scale cluster and workloads. (3) Finally, the simulator needs to be further optimized in terms of execution rate.

## Figures and Tables

**Table 1:** Job scheduling scenarios of evaluation.

Scenario	Job Scheduling Scenarios		
1	TaskQueue workloads (driven by Alibaba trace 2018)	Daytime	Original resources
2			50% decrease
3			25% decrease
4			25% increase
5			50% increase
6		Night	Original resources
7			50% decrease
8			25% decrease
9			25% increase
10			50% increase
11	JobQueue workloads (driven by Alibaba trace 2020)	Daytime	Original resources
12			50% decrease
13			25% decrease
14			25% increase
15			50% increase
16		Night	Original resources
17			50% decrease
18			25% decrease
19			25% increase
20			50% increase

**Table 2:** Real and simulation running times under TaskQueue workloads and changeable resources.

Scenario	Real Running Time (s)/Simulation Running Time (s)										
	BRA	EP	ILP	IPAP	LRP	MRP	NAP	NLP	NPAPP	RLP	TTP
1	1868.64/43.1	1987.29/49.6	2214.69/46.3	1947.74/49.5	1927.97/43.2	1680.79/44.1	2026.84/43.8	1779.66/43.6	2026.84/43.5	1908.19/43.7	1987.29/42.9
2	2422.32/52.15	2402.54/60.02	2501.41/56.02	2491.52/59.9	2521.19/52.27	2214.69/53.36	2629.94/53	2521.19/52.76	2669.49/52.64	2461.86/52.88	2600.28/51.91
3	1898.3/47.52	2076.27/54.68	1838.98/51.05	2115.82/54.57	1977.4/47.63	1789.55/48.62	2155.37/48.29	2214.69/48.07	1937.85/47.96	2224.58/48.18	2016.95/47.3
4	1344.63/40.95	1621.47/47.12	1562.15/43.99	1601.69/47.03	1670.9/41.04	1265.54/41.9	1661.02/41.61	1819.21/41.42	1334.75/41.33	1888.42/41.52	1572.03/40.76
5	1255.65/38.79	1601.69/44.64	1532.49/41.67	1542.37/44.55	1572.03/38.88	1166.67/39.69	1443.5/39.42	1740.11/39.24	1502.82/39.15	1700.56/39.33	1552.26/38.61
6	4745.76/79.2	5190.68/81.6	5170.9/76.2	5131.35/77.1	5437.85/74.7	4874.29/82.5	5151.13/76.2	5022.6/79.5	5121.47/80.1	5091.81/77.1	5131.35/79.5
7	5411.86/109.06	5778.95/112.36	5813.56/104.93	5709.74/106.17	6242.65/102.86	5668.22/113.6	5716.66/104.93	5931.21/109.47	5778.95/110.3	5592.09/106.17	5772.03/109.47
8	4933.61/89.73	4903.95/92.45	4775.42/86.33	4933.61/87.35	4963.27/84.64	4686.44/93.47	4864.4/86.33	4725.99/90.07	4903.95/90.75	5230.22/87.35	5200.56/90.07
9	3638.42/76.03	3608.76/78.34	3816.38/73.15	3638.42/74.02	4399.72/71.71	3549.43/79.2	3875.7/73.15	3569.21/76.32	3905.37/76.9	3875.7/74.02	3707.63/76.32
10	3440.68/72.07	3549.43/74.26	3638.42/69.34	3391.24/70.16	4201.98/67.98	3351.69/75.08	3539.55/69.34	3588.98/72.35	3648.3/72.89	3509.89/70.16	3588.98/72.35

**Table 3:** Real and simulation running times under JobQueue workloads and changeable resources.

Scenario	Real Running Time (s)/Simulation Running Time (s)												
	DRF_BRA	DRF_LRP	DRF_MRP	GANG_BRA	GANG_DRF_ BINPACK	GANG_DRF_ BRA	GANG_DRF_ LRP	GANG_DRF_ MRP	GANG_LRP	GANG_MRP	SLA_BRA	SLA_LRP	SLA_MRP
11	2891.5/119.2	2662.8/119.6	2471.7/118.4	2628.3/118	2565.7/120	2806.9/120.4	2349.5/118.8	2778.7/119.2	2913.4/126.8	2427.8/120.4	2853.9/119.2	2597/118.4	2622/122
12	3984.8/131.1	4144.5/131.6	3980.1/130.2	3980.1/129.8	3984.8/132	3984.8/132.4	4144.5/130.7	3984.8/131.1	4144.5/139.5	3984.8/132.4	3984.8/131.1	4153.9/130.2	3975.4/134.2
13	3345.7/124	3402.1/124.4	3063.8/123.1	3336.3/122.7	3049.7/124.8	3341/125.2	3406.8/123.6	3063.8/124	3402.1/131.9	3063.8/125.2	3336.3/124	3402.1/123.1	3059.1/126.9
14	2250.8/113.2	2086.4/113.6	2664.3/112.5	2250.8/112.1	2669/114	2250.8/114.4	2081.7/112.9	2659.6/113.2	2086.4/120.5	2669/114.4	2250.8/113.2	2086.4/112.5	2664.3/115.9
15	1964.2/107.3	1931.3/107.6	2546.9/106.6	1973.6/106.2	2546.9/108	1964.2/108.4	1926.6/106.9	2542.2/107.3	1931.3/114.1	2542.2/108.4	1973.6/107.3	1926.6/106.6	2537.5/109.8
16	7581.1/192.6	6954.5/193.2	7437/179.4	7158.2/192.6	7565.4/181.2	7631.2/193.2	6907.5/191.4	7931.9/192	7336.7/190.8	7784.7/178.8	7352.4/190.2	7001.5/178.8	7001.5/187.2
17	9961.9/249.6	10361.3/250.4	9950.2/232.5	9950.2/249.6	9961.9/234.8	9961.9/250.4	10361.3/248.1	9961.9/248.8	10361.3/247.3	9961.9/231.7	9961.9/246.5	10384.8/231.7	9938.4/242.6
18	8364.2/218.2	8505.2/218.9	9144.3/203.3	8340.7/218.2	8282.5/205.3	8352.5/218.9	8517/216.9	8315.4/217.5	8505.2/216.2	8322/202.6	8340.7/215.5	8505.2/202.6	8308.8/212.1
19	5627.1/184.9	5545.8/185.5	6660.9/172.2	5627.1/184.9	6672.6/174	5627.1/185.5	5532.6/183.7	6649.1/184.3	5361.6/183.2	6672.6/171.6	5627.1/182.6	5776/171.6	6660.9/179.7
20	5239.4/175.3	5157.2/175.8	6249.7/163.3	5262.9/175.3	5624.7/164.9	5239.4/175.8	5145.4/174.2	6381.3/174.7	5157.2/173.6	6512.8/162.7	5262.9/173.1	5145.4/162.7	6447/170.4

**Table 4:** Reductions in running time under TaskQueue workloads and changeable resources.

Scenario	Reductions (Real Running Time/Simulation Running Time)										
	BRA	EP	ILP	IPAP	LRP	MRP	NAP	NLP	NPAPP	RLP	TTP
1	43.36×	40.07×	47.83×	39.35×	44.63×	38.11×	46.27×	40.82×	46.59×	43.67×	46.32×
2	46.45×	40.03×	44.65×	41.6×	48.23×	41.5×	49.62×	47.79×	50.72×	46.56×	50.09×
3	39.95×	37.97×	36.03×	38.77×	41.52×	36.81×	44.63×	46.07×	40.41×	46.17×	42.64×
4	32.84×	34.41×	35.52×	34.06×	40.71×	30.21×	39.92×	43.92×	32.3×	45.49×	38.57×
5	32.37×	35.88×	36.78×	34.62×	40.43×	29.39×	36.62×	44.35×	38.39×	43.24×	40.2×
6	59.92×	63.61×	67.86×	66.55×	**72.8**×	59.08×	67.6×	63.18×	63.94×	66.04×	64.55×
7	49.62×	51.43×	55.41×	53.78×	60.69×	49.9×	54.48×	54.18×	52.39×	52.67×	52.73×
8	54.98×	53.04×	55.31×	56.48×	58.64×	50.14×	56.34×	52.47×	54.04×	59.87×	57.74×
9	47.85×	46.07×	52.17×	49.16×	61.35×	44.82×	52.98×	46.77×	50.79×	52.36×	48.58×
10	47.74×	47.8×	52.47×	48.34×	61.81×	44.64×	51.04×	49.61×	50.05×	50.03×	49.61×

**Table 5:** Reductions in running time under JobQueue workloads and changeable resources.

Scenario	Reductions (Real Running Time/Simulation Running Time)												
	DRF_BRA	DRF_LRP	DRF_MRP	GANG_BRA	GANG_DRF_ BINPACK	GANG_DRF_ BRA	GANG_DRF_ LRP	GANG_DRF_ MRP	GANG_LRP	GANG_MRP	SLA_BRA	SLA_LRP	SLA_MRP
11	24.26×	22.26×	20.88×	22.27×	21.38×	23.31×	19.78×	23.31×	22.98×	20.16×	23.94×	21.93×	21.49×
12	30.4×	31.49×	30.57×	30.66×	30.19×	30.1×	31.71×	30.4×	29.71×	30.1×	30.4×	31.9×	29.62×
13	26.98×	27.35×	24.89×	27.19×	24.44×	26.69×	27.56×	24.71×	25.79×	24.47×	26.91×	27.64×	24.11×
14	19.88×	18.37×	23.68×	20.08×	23.41×	19.67×	18.44×	23.49×	17.31×	23.33×	19.88×	18.55×	22.99×
15	18.31×	17.95×	23.89×	18.58×	23.58×	18.12×	18.02×	23.69×	16.93×	23.45×	18.39×	18.07×	23.11×
16	39.36×	36×	41.45×	37.17×	41.75×	39.5×	36.09×	41.31×	38.45×	43.54×	38.66×	39.16×	37.4×
17	39.91×	41.38×	42.8×	39.86×	42.43×	39.78×	41.76×	40.04×	41.9×	42.99×	40.41×	44.82×	40.97×
18	38.33×	38.85×	**44.98**×	38.23×	40.34×	38.16×	39.27×	38.23×	39.34×	41.08×	38.7×	41.98×	39.17×
19	30.43×	29.9×	38.68×	30.43×	38.35×	30.33×	30.12×	36.08×	29.27×	38.88×	30.82×	33.66×	37.07×
20	29.89×	29.34×	38.27×	30.02×	34.11×	29.8×	29.54×	36.53×	29.71×	40.03×	30.4×	31.63×	37.83×

## Data Availability

The data and materials used to support the findings of this study are available from the corresponding author upon request.
